# CASM-AMFMNet: A Network Based on Coordinate Attention Shuffle Mechanism and Asymmetric Multi-Scale Fusion Module for Classification of Grape Leaf Diseases

**DOI:** 10.3389/fpls.2022.846767

**Published:** 2022-05-24

**Authors:** Jiayu Suo, Jialei Zhan, Guoxiong Zhou, Aibin Chen, Yaowen Hu, Weiqi Huang, Weiwei Cai, Yahui Hu, Liujun Li

**Affiliations:** ^1^College of Computer and Information Engineering, Central South University of Forestry and Technology, Changsha, China; ^2^Plant Protection Research Institute, Hunan Academy of Agricultural Sciences (HNAAS), Changsha, China; ^3^Department of Civil, Architectural and Environmental Engineering, Missouri University of Science and Technology, Rolla, MO, United States

**Keywords:** CASM-AMFMNet, coordinate attention shuffle mechanism asymmetric, multi-scale fusion module, grape leaf diseases, GSSL, image enhancement

## Abstract

Grape disease is a significant contributory factor to the decline in grape yield, typically affecting the leaves first. Efficient identification of grape leaf diseases remains a critical unmet need. To mitigate background interference in grape leaf feature extraction and improve the ability to extract small disease spots, by combining the characteristic features of grape leaf diseases, we developed a novel method for disease recognition and classification in this study. First, Gaussian filters Sobel smooth de-noising Laplace operator (GSSL) was employed to reduce image noise and enhance the texture of grape leaves. A novel network designated coordinated attention shuffle mechanism-asymmetric multi-scale fusion module net (CASM-AMFMNet) was subsequently applied for grape leaf disease identification. CoAtNet was employed as the network backbone to improve model learning and generalization capabilities, which alleviated the problem of gradient explosion to a certain extent. The CASM-AMFMNet was further utilized to capture and target grape leaf disease areas, therefore reducing background interference. Finally, Asymmetric multi-scale fusion module (AMFM) was employed to extract multi-scale features from small disease spots on grape leaves for accurate identification of small target diseases. The experimental results based on our self-made grape leaf image dataset showed that, compared to existing methods, CASM-AMFMNet achieved an accuracy of 95.95%, F1 score of 95.78%, and mAP of 90.27%. Overall, the model and methods proposed in this report could successfully identify different diseases of grape leaves and provide a feasible scheme for deep learning to correctly recognize grape diseases during agricultural production that may be used as a reference for other crops diseases.

## Introduction

Grape is a popular fruit worldwide with multiple nutritional components. The active compounds in grape extracts have antioxidant, antibacterial, anti-inflammatory, and anti-carcinogenic activities and thus utilized to generate products that can alleviate and treat hypertension (Sabra et al., 2021). The continuous improvement of living standards and high demand for grapes have been important driving factors in the progressive development of the grape planting industry and growing areas of grape cultivation over recent years. However, grapes are easily susceptible to weather, environmental variables, insect pests, bacteria, and fungi during the cultivation process (Ampatzidis et al., 2017), with frequent risk of black rot, black measles, leaf blight, downy mildew, and other grape leaf diseases that seriously affect growth and contribute significantly to reduction of grape quality and yield, resulting in huge financial losses to farmers.

Infection patterns of grape diseases are usually manifested on the leaves (Chouhan et al., 2020), which can be easily collected and examined to characterize diseased spots. Traditionally, grape leaf diseases are evaluated *via* visual inspection by fruit farmers and plant protection experts (Pound et al., 2017), which is associated with problems of strong subjectivity, slow speed, a high misidentification rate, poor real-time performance, and high dependence on advice by experts (Bock et al., 2010). Since grape leaves display small spot areas in the early stages of disease, manual detection is difficult. In addition, when collecting grape leaf images in the natural environment, some disease spots of the leaves are obscured, resulting in fewer details of features that are identifiable. Evaluation of leaf disease *via* visual inspection is a considerable challenge (Chouhan et al., 2018). However, accurate early identification of the symptoms of grape disease and effective control spread should aid in successfully minimizing losses. Therefore, timely and efficient machine learning methods to identify the disease spots of grape leaves are extremely helpful for farmers to rapidly assess the disease type and extent of infestation. Appropriate prevention and control can reduce the impact of disease, in turn, improving the yield and quality of grapes and safeguarding the economic benefits of fruit farming. At present, three major problems exist in identification of grape leaf diseases. (1) Imaging of grape leaf has issues of edge blurring and noise. For instance, among the grape leaf images we obtained, inconspicuous contrast, blurred edges, and noise were prevalent, which affect leaf recognition by the network, and in severe cases, the recognition and extraction of disease features, leading to inaccurate classification of grape leaf diseases. (2) Images have background interference. When analyzing grape leaf images, shape, size, and color of spots of different diseases are usually extracted. However, complex backgrounds can affect feature extraction. The network extracts the interference factors in the background as features, leading to inaccurate classification. (3) Grape leaf disease spots are extremely small. Since the grape leaves are relatively small and some disease spots themselves are minute at the beginning, small and dense disease spots may also appear on the same leaf, making detection difficult and leading to lack of extracted feature information. Consequently, misclassification of different grape leaf diseases is relatively common.

To solve the problem of blurred edges and noise in grape leaf images, Liu et al. (2018) proposed a novel adaptive-rendering approach based on feature reconstruction to eliminate Monte Carlo noise while preserving image details. However, the edge information of images obtained with this method becomes blurred. Clinton (2017) used Sobel algorithms to detect the edges of blurred images, which improved image quality and facilitated restoration, but the image edges detected with this method were discontinuous, and the lines were thick, resulting in loss of some edge details. Cruz et al. (2017) applied a small-window median filter to remove noise in the leaf image dataset. This method effectively preserved the sharp edges of plant leaves, but the effect of Gaussian noise removal in the background was not ideal. In this study, the Gaussian filters Sobel smooth de-noising Laplace operator (GSSL) algorithm was applied to preprocess the image and process the grape leaf image using multiple steps, including ideal high-pass filter, Sobel operator, and smooth filter. The images obtained exhibited clear edges, obvious contrast, and less noise. At the same time, the texture features of diseased grape leaves were preliminarily enhanced.

To resolve the problem of image background interference, Gao and Lin (2019) proposed an accurate and fully automatic segmentation method for medicinal plant leaf imaging under complex backgrounds. However, this method was not successful when applied to gray images. An algorithm combining simple linear iterative cluster (SLIC) with support vector machine (SVM) was proposed by Sun and colleagues (Huang et al., 2018) to extract a saliency map of tea leaf disease under complex backgrounds (Sun et al., 2019). This procedure uses simple linear iterative clustering for preprocessing to separate significant regions from the background. However, errors can occur when separating the background and disease regions, resulting in loss of a number of the features at the preprocessing stage.

For the problem of small leaf disease spots, Liu et al. (2020a) proposed improved deep convolutional neural networks based on convolutional neural network (CNN) for grape leaf disease recognition using depthwise separable convolution to establish the first two convolutional layers, designated DICNN. Deep separable convolution is used to reduce the model parameters and over-refinement. Next, the concept structure is employed to improve the extraction performance of multi-scale convolution for disease points. Finally, the dense connection strategy is introduced to promote the fusion of multidimensional features between the concept structures for alleviating the problem of gradient disappearance and facilitating feature reuse and propagation. However, when the simplest CNN is used as the model backbone, the gradient descent algorithm can be easily applied to make the training results converge to the local minimum rather than the global minimum. The pooling layer loses considerable valuable information and overlooks the correlation between the local and global layers. On the other hand, in a complex environment, precise disease location of grape leaves is not achieved and the disease can easily be confused with a similar background, resulting in reduced accuracy of identification. The use of deep separable convolution significantly reduces the model parameters but simultaneously decreases the model capacity, leading to lower accuracy of disease recognition. The presence of accumulating perception structures also increases the difficulty of using the model in downstream tasks and amount of calculation. Xie et al. (2020) proposed a rapid DR-IACNN with higher feature extraction capability to identify grape leaves based on the detection algorithms of GLDD and fast R-CNN. Firstly, the use of Resnet improved the backbone. A double RPN structure was designed to achieve better feature extraction of small lesions through upsampling and downsampling. Disease features were extracted by introducing the inception-v1 and inception-ResNet-v2 modules and Se blocks to obtain further features. While this method facilitates network focus on the diseased points of grape leaves rather than the background, drawbacks of the Resnet model include a large number of parameters and high volume of the model after training. Despite the increased accuracy of identification of diseases from grape leaf images, several problems, such as large network parameters, complex calculations, and poor real-time performance, remain to be resolved. SE blocks only consider reweighting the importance of each channel by simulating the channel relationship while disregarding location information, consistently resulting in significant classification errors for grape leaf diseases.

In view of the above issues, we proposed CASM-AMFMNet based on CoAtNet to improve the identification and classification of grape leaf diseases. Firstly, CoAtNet effectively combined the convolution and attention layers to achieve a better balance between recognition accuracy and efficiency and showed better generalization ability and capacity of the network model. As a backbone, CoAtNet initially extracted the local edge features of disease images, such as contour and color. The CASM module was effectively used to solve the problems existing in the traditional SE block, embed location information into the channel attention system so that the network could perform over a wide range, and avoid computational overheads to accurately locate, capture, and extract feature information used to distinguish between diseases and reduce the interference of complex background information. Finally, the AMFM module, which could process the input image position and semantic information on different scales that were then rescaled and combined with the module input, was introduced to extract multi-scale features of small targets. Our model effectively reduced the quantity of calculations and training time of the network.

The main contributions of this study are as follows:

1. A new algorithm GSSL is proposed to enhance grape leaf imaging. The method initially grayscales the image and subsequently processes high-pass filtering and the grayscale image using the Sobel operator to obtain the mask. Simultaneously, the grayscale image is smoothed and denoised, and the image obtained is processed using the Laplace operator to enhance grape leaf details. Finally, a preliminary texture-enhanced grape leaf image is generated using this image and the mask.
2. To reduce the background interference in grape leaf images and improve the extraction of small disease spots, we have proposed a new network, CASM-AMFMNet. The design is as follows: (a) A coordinate attention shuffle mechanism (CASM) suitable for retaining accurate disease location information along two different spatial directions, and capturing the domain of interest is utilized to extract feature information for disease discrimination. The module uses the input grape leaf disease image feature maps to perform group convolution (GC), and each sub-feature map captures specific semantic information on network training. Meanwhile, adding a channel shuffle at the end of the module can effectively improve the correlations between different channels in the group convolution and integrate feature information on each channel, improve the network fit, and merge the extracted image features with fewer numbers of parameters to obtain higher model accuracy. The module assigns weights to the feature maps according to different semantic information. The weight of the channel in which the grape leaf disease features are located is the largest, which effectively suppresses the interference of analogs and extracts disease features under complex background interference. (b) An asymmetric multi-scale fusion module (AMSM) was designed, which assigns multi-scale perceptual fields in the main network and effectively extracts details such as the shape and contour of small grape leaf disease spots. The ACB on each branch can enhance the robustness of the model to flipped or rotated images, improve training accuracy, and further reduce the number of parameters and computational efforts of the model. The module may be used to better focus on smaller spots that are easily overlooked when performing feature extraction and extract features while improving the precision of small target extraction and reducing the training time required by the network.
3. Our method achieved an accuracy of 95.95% in identification of five grape leaf samples, F1 score of 95.78%, and mAP of 90.27%. Furthermore, the model had a good discriminatory power for distinguishing between healthy and diseased leaves, facilitated classification of grape leaves in complex environments, and effectively extracted small disease spot targets. This technique could also be used with good results in public datasets. Rapid and accurate identification and classification of leaf diseases should effectively reduce loss of grape production in agriculture.

## Related Work

**FIGURE 1:**
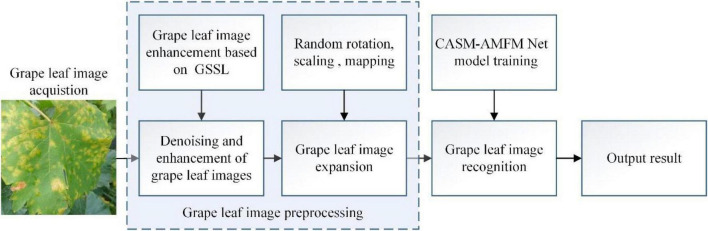
A working principle diagram of the system.

To reduce the harmful effects of diseases in plants, many experts and scholars have recently focused on exploring the utility of artificial intelligence in identifying and classifying plant diseases rapidly and effectively. These studies have made significant contributions to the recognition of plant diseases, especially grape leaf diseases. For instance, Kundu et al. (2021) developed the framework of “automatic and intelligent data collector and classifier” by integrating IoT and deep learning to precisely predict blast and rust diseases in pearl millet. Padol and co-workers used the SVM classification technique for grape leaf diseases. The K-means clustering segmentation algorithm was initially used to identify the region of disease and extract texture and color features, followed by a classification technique for stratification of leaf disease categories (Padol and Yadav, 2016). This method showed an accuracy of 88.89%. The group of Narvekar used the SGDM matrix method to analyze grape leaf diseases and systematically discussed effective methods for disease detection *via* leaf feature inspection (Narvekar et al., 2014). Their method was able to achieve accurate disease detection with little computational effort. Peng et al. (2021) performed extraction with CNN plus support vector machine (SVM) to diagnose grape leaves based on fused deep features. Using this method, the SVM classifier could be trained to achieve the same classification accuracy as the CNN model. Leaf GAN utilized by Liu et al. (2020b) to analyze four different grape leaf disease images successfully overcame the overfitting problem and improved identification accuracy. Jaisakthi and colleagues further used different machine learning techniques such as SVM, AdaBoost, and Random Forest tree (Liu et al., 2020b), to identify grape leaf diseases. Their results showed that SVM was able to achieve 93% accuracy (Jaisakthi et al., 2019). Each of the above methods has its own merits, and network models using SVM classifier and CNN clearly have the ability to successfully classify grape leaf diseases. However, identification of grape leaf diseases needs to be optimized in many areas to achieve higher classification accuracy. In addition, grape leaf images with blank backgrounds have mainly been used as the datasets for experiments to date, which are conducive to classification of simple images and less suitable for complex backgrounds. Meanwhile, the current network is inadequate for recognition of small target diseases and leads to generation of errors. Here, we propose a model better adapted to extract features of grape leaf small target diseases under complex backgrounds based on CASM-AMFMNet. The specific scheme of grape leaf disease identification and classification is presented in Figure 1.

## Materials and Methods

### Data Acquisition

Grape leaf data used in this study were classified into five categories: (1) healthy, (2) black rot, (3) black measles, (4) leaf blight, and (5) downy mildew. The dataset was mainly derived from two sources. One part was collected from the Tianlu vineyard (Changsha, China), which incorporated images of healthy, black rot, leaf blight, and downy mildew leaves from different periods taken on both sunny and cloudy days. We constantly changed the shooting angles and distances and collected grape leaf images of different colors, sizes, and backgrounds. To ensure accuracy of recognition, the grape leaves filled the image to the maximum extent. The other part of the dataset included leaves of different grape varieties with black measles along with the above diseases from a complex environment, comprising several orchards located using websites such as Kaggle (2021) and Google, among which 2,603 images were screened. Using the available information and by consulting relevant scholars, we reorganized and reclassified the collected images, screened those that were categorized, and deleted blurred images. Ultimately, 3,409 grape leaf images were collected from both dataset sources. The numbers and ratios of different categories of grape leaf images are shown in Table 1.

**TABLE 1:** Number and proportion of grape leaf images.

Category	Example	Number (Before)	Proportion/ % (Before)	Number (After)	Proportion/ % (After)
Healthy		814	23.88	3,166	20.02
	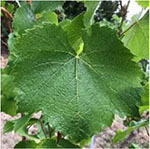				
Black rot		725	21.27	3,148	19.89
	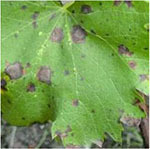				
Black Measles		669	19.62	3,175	20.06
	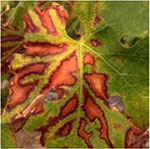				
Leaf blight	,	674	19.77	3,154	19.93
	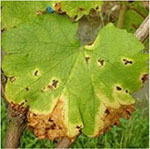				
Downy Mildew		527	15.46	3,181	20.10
	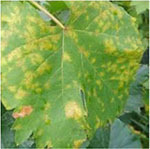				

Five types of grape leaves were analyzed in this study. Healthy grape leaves were dark green and palm shaped with a surface free of disease spots and clear veins. Black rot is a fungal disease (Tomoiaga and Chedea, 2020) usually occurring at the leaf margin. After their appearance, disease spots gradually expand to circular spots that are gray-white in the center and brown on the outer edge, with a grayish-brown margin. At the later stages of the disease, small dots arranged in a ring appear on the disease spots. During early infection with black measles caused by fungal complexes, such as Phaeoacremonium (Nerva et al., 2019), light green spots are formed between leaf veins that continue to expand to the end of branches, and eventually become tiger striated. At the initial disease stage, leaf blight caused by fungi, such as Pestalotiopsis (Nuthan et al., 2021), presents as light-brown, irregular, and angular small spots, which then expand into circular or oval brown spots with a brown or tan center and a dark brown margin with a water-stained outer edge. Downy mildew [caused by *Plasmopara viticola* (Berk. & Burt.) Berl. & De Toni belonging to the order Peronosporales, a pathogen of grape-specific oomycetes (Fawke et al., 2015)] produces small, indistinct, yellowish watery spots with indistinct edges in the early stages of infection, which gradually expand into light green or yellow-brown spots on the front of leaves. Images of individual grape leaf diseases clearly show distinct spot characteristics. However, black rot and leaf blight have relatively similar features. Some leaf images show many tiny spots in both the early and later stages of infection, and therefore, extraction of their specific characteristics is important for disease recognition and management.

**FIGURE 2:**
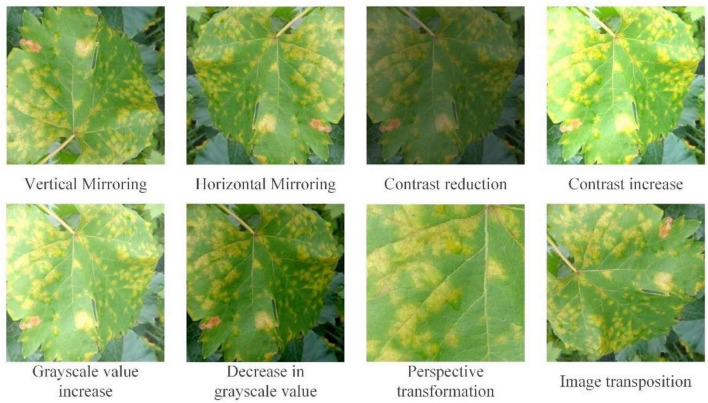
Eight transformation images of downy mildew as an example.

Convolutional neural networks require a large number of samples for model training, and acquisition of large quantities of disease images is a considerable challenge. Therefore, we expanded the dataset in this study using image transformation algorithms (Ghosal et al., 2018) to increase the sample number, prevent overfitting in the network, and improve the performance of the model (Pawara et al., 2017; Barbedo, 2018). We employed the algorithms of perspective transformation, geometric transformation (Sladojevic et al., 2016) [e.g., horizontal and vertical mirroring flip (Wang et al., 2017)], and intensity transformation (e.g., contrast increase and decrease and brightness enhancement and decrease) (Khan et al., 2018) to increase the number of grape disease images with a view to simulating the real collection environment and improving diversity and accuracy. With the aid of “vertical mirroring,” “horizontal mirroring,” “contrast reduction by 10%,” “contrast increase by 10%,” “Grayscale value increase by 45,” “Grayscale value reduction by 45,” “perspective transformation,” and “image transposition” processes, grape leaf diseases were imaged. Taking the grape leaf downy mildew image as an example, the eight transformed images are shown in Figure 2. Original downy mildew leaves are usually light green. The disease spots could be enhanced by adjusting the contrast of disease spots and leaf colors. Through perspective transformation, the disease spot could be enlarged, which facilitated observation of the water stain shape. Different angle transformation methods were utilized to examine the shapes of the diseased leaves from different angles. At the same time, the brightness transformation simulated leaf images in different environments, leading to enhancement of disease characteristics.

### Evaluation Indicators

To determine the effectiveness of our method and quantitatively analyze the accuracy of grape leaf image recognition and classification, evaluation criteria used on the one hand were accuracy Equation (1), precision Equation (2), recall Equation (3), and mAP Equation (4) for assessment of the model performance. On the other hand, considering the limitations of storage and computational power during network operation, FPS (the number of grape leaf images recognized by the model per second, representing speed of detection), recognition time used per batch of images, param, MFLOPs, and FLOPs were also used as criteria for model evaluation.


(1) $$A\,c\,c\,u\,r\,a\,c\,y=\frac{T\,F+T\,P}{F\,P+T\,N+T\,P+F\,N}$$

(2) $$\mathrm{Pre}\,c\,i\,s\,i\,o\,n=\frac{T\,P}{T\,P+F\,P}$$

(3) Re⁢c⁢a⁢l⁢l=T⁢PT⁢P+F⁢N

(4) $$m\,A\,P=\int_{0}^{1}P\,\left(R\right)\,dR$$

*TP* indicates the number of accurately identified grape leaf disease categories, *TN* the number of incorrectly identified non-grape leaf diseases, *FP* the number of correctly identified non-grape leaf diseases, and *FN* the number of grape leaf diseases that were not correctly identified. Precision indicates the proportion of all correctly predicted grape leaf images to the number of true correct samples and incorrectly predicted correct samples within the data. Recall signifies the proportion of grape leaf images of all predicted correct samples in relation to all true correct samples. For comprehensive evaluation of the model, the harmonic average F1 score of precision and recall was applied as the evaluation index, as shown in Equation (5).


(5) $$F\,1=\frac{2*p\,r\,e\,c\,i\,s\,i\,o\,n*r\,e\,c\,a\,l\,l}{p\,r\,e\,c\,i\,s\,i\,o\,n+r\,e\,c\,a\,l\,l}$$

FPS representing the number of images detected by the model per second (speed of detection) can be obtained from Equation (6) below.


(6) $$F\,P\,S=\frac{N}{T}$$

In Equation (6), *N* represents the number of recognized samples and *T* the time required to test all samples.

For the evaluation index of the image quality, we selected grayscale mean, peak signal-to-noise ratio (PSNR), and entropy to quantitatively analyze the quality of image enhancement and compare with the visual effects. The grape leaf image with a high mean gray value is bright overall, which is easier to identify than an image with a low mean gray value. Larger PSNR corresponds to lower distortion of the grape leaf image. Larger entropy values are correlated with richer texture information. Mean, PSNR, and entropy are calculated using Equations (7–9).


(7) $$m\,e\,a\,n=\frac{1}{X\times Y}\,\sum_{i=1}^{X}\sum_{j=1}^{Y}R\,\left(i,j\right)$$

(8) $$P\,S\,N\,R=10\times \mathrm{lg}\left(\frac{M\,A\,X^{2}}{M\,S\,E}\right)$$

(9) $$e\,n\,t\,r\,o\,p\,y=\sum_{i=0}^{225}P\,\left(i\right)\times \log_{2}p\,\left(i\right)$$

Here, X × Y represents the total number of pixels in the image, *R*(*i*,*j*) is the pixel value of the image point (*i*,*j*), *R*(*i*,*j*), and *f*(*i*,*j*) are grayscale values of the output and input images at point (*i*,*j*), respectively, *MSE* is the mean square error, and 255 is the maximum gray level. *P*(*i*) denotes the proportion of pixels with gray value *i* to total pixel number.

### Gaussian Filters Sobel Smooth De-Noising Laplace Operator

The images of grape leaves in the dataset have a number of issues, such as inconspicuous contrast, blurred edges, and noise. Therefore, the acquired grape leaf images need to be pre-processed *via* filtering, noise reduction, and enhancement.

We have proposed a GSSL algorithm to denoise and enhance grape leaf images in this study. Compared with the traditional preprocessing method, our procedure does not need to segment the background and leaves but deepens the edge contours of grape leaves, reduces image noise, and uses multi-step combination processing, such as ideal high-pass filter, Sobel operator, and smooth filter. Useful information from the image is extracted to the maximum extent possible and the noise reduced. Through image superposition, the authenticity of the original image is retained, and image distortion is effectively prevented while highlighting useful information. The image is obtained as *E*(*i*,*j*), as shown in the Equation (10).


$$E\left(i,j\right)=\{\sqrt{s_{i}^{2}+s_{j}^{2}}\times [\sum_{m=-1}^{1}\sum_{n=-1}^{1}k\left(m,n\right)p\left(i-m,j-n\right)$$

(10)       +f(i,j)]}+f(i,j)

Here, *k*(*m,n*) is the Laplace operator mask of 3×3, *p*(*i,j*) the gray value after smooth filtering, *s*_*i*_ and *s*_*j*_ the gradients of the image in the horizontal and vertical directions, respectively, and *f*(*i,j*) the gray value of the input image at point (*i,j*). The specific workflow of the GSSL algorithm is as follows:

Step 1: The data of grape leaf images are normalized. Color images are converted into grayscale, and the normalized and grayscale-processed grape leaf images used as the input for subsequent steps.

Step 2: The mask required is obtained and a simple detailed enhancement image acquired in two steps.

1. First, the ideal high-pass filter is used to process the input image. Through this step, the high-frequency part of the grape leaf image in the frequency domain space, i.e., edge details, can be extracted. Next, the image extracted with the ideal high-pass filter is added to the input image to obtain a simple edge enhancement image. The Sobel operator is subsequently used as the convolution kernel for the convolution operation on the image obtained in the previous step to acquire edge information for use as a mask.
2. The input grape leaf image is smoothed, denoised, and processed with the Laplace operator to highlight minor details. Incorporation of this result into the input image generates a preliminary detail-enhanced image.

**FIGURE 3:**
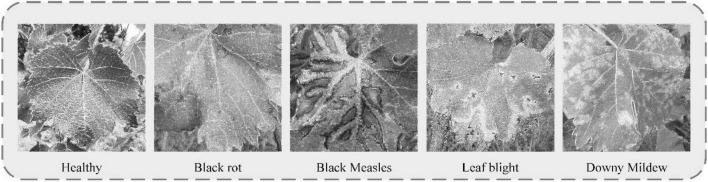
A Gaussian filters Sobel smooth de-noising Laplace operator (GSSL) enhancement effect chart for five grape leaves.

Step 3: Image calculation. The mask image processed in Step 1 is multiplied by the initial enhanced image obtained in Step 2 for efficient extraction of edge and detailed information from the grape leaf image. The input image is then added to obtain an enhanced grape leaf image. A representative enhanced image obtained with the GSSL algorithm is shown in Figure 3.

As observed from the figure, grape leaf image processed using GSSL displays a certain shape of dark spots with an obvious edge contour. For example, “black measles” and “leaf bright” can clearly be utilized to detect the location of the spots. Although the disease contour is not obvious, colors of spots are easily distinguishable from the healthy leaf surface. The veined texture of all enhanced grape leaves is also more prominent, weakening the background-independent factors and reducing noise in the image, which increases the convenience of subsequent extraction of grape leaf characteristics by the neural network model.

### Coordinated Attention Shuffle Mechanism-Asymmetric Multi-Scale Fusion Module Net

In the images of grape leaf disease we collected, most of the grape leaves that have diseases show background interference. This entails that the whole network is vulnerable to impeded recognition, resulting in the incorrect localization of the identified disease areas. In addition, some of the diseased areas are almost integrated with the grape branches in the background, or their leaf shapes and contour after leaf curling are easily confused with the shapes and contours of the flowers in the background, resulting in recognition errors, which reduces the recognition accuracy of the grape leaf diseases.

Therefore, the reduction of the impact of a complex background on disease recognition and the realization of the feature extraction of small disease spots of grape leaf diseases are problems that need urgent solution. In response, this paper designs a CASM-AMFMNet for grape leaf disease identification and classification. First, the network proposes a backbone based on CoAtNet. Then, the CASM module is used to accurately capture location information for grape leaf diseases and to focus on their essential features to reduce complex background information. Finally, AMFM is used to give the main network multi-scale perceptual fields, extracting subtle features such as disease spot shapes and contours in all directions as much as possible to improve the accuracy of the network recognition of small targets, effectively reducing the amount of parameter computation and reducing the training time of the network.

**FIGURE 4:**
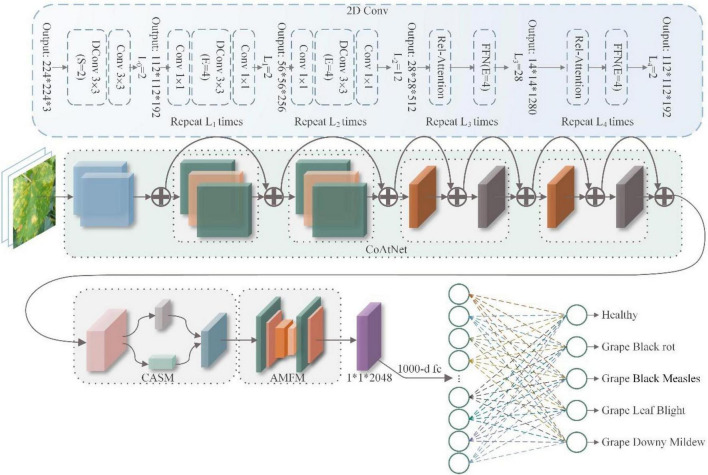
Coordinated attention shuffle mechanism-asymmetric multi-scale fusion module net (CASM-AMFMNet) structure.

The overall structure of the CASM-AMFMNet is shown in Figure 4, which is mainly divided into the following three parts:

1. In the first part, we used CoAtNet as the backbone. It uses convolution for downsampling up to stride = 16 to perform preliminary extraction of the features of grape leaf disease and bring about higher accuracy in the network, better generalization, and larger capacity.

The second part consists of a CASM module and an AMFM. First, the CASM divides the feature map into G groups (see the following text for the definition of G). Then, we used coordinate attention mechanism (CAM) for an average pool of the horizontal and vertical directions; this process assigns different weights to channel and spatial features, suppresses background information that is invalid with respect to the features of grape leaf disease, captures the accurate location information on the disease, and enhances the expressiveness of the network. Next, through three 1*1 convolutions, a feature map of the same size and enhanced representation as the input grape leaf feature image is obtained. Finally, the feature maps obtained from the first layer are added to the module after the attention mechanism for the channel shuffle to enhance the expression of the learned features and use the SELU activation function to enhance the nonlinear expression capability of the network. We added the CASM module at the backbone end of the model to fully consider the global and local texture features of grape leaf disease. AMFM consists of two 1*1 convolutions and *n* 3*3 convolutions at different scales, and the feature fusion of the disease features extracted by backbone can strengthen the recognition capability of small disease targets. The convolution adopts ACB convolution, which is done to reduce the amount of parameter computation and speed up network training.

2. In the third part, the global pooling downsampling layer is connected to the fully connected layer. Finally, the output is transformed into a probability distribution using Softmax to obtain the classification results of grape leaf disease images.

The following three subsections elaborate on the network.

#### CoAtNet

ConvNet has good generalization capability and rapid convergence speed. Nevertheless, its perceptual range is limited by the size of the convolution kernel, while its large-scale perceptual ability is conducive to the model to obtain additional contextual information. A transformer tends to have a larger model capacity, but its generalization capability is poor relative to that of ConvNet due to the lack of correct induction deviation. Therefore, this paper uses CoAtNet (Dai et al., 2021) as the backbone, which effectively combines ConvNet with a transformer to achieve a better trade-off between accuracy and efficiency, and its backbone network uses residual connections. As a result, the network structure has sufficient depth to retain additional feature information and facilitate the fusion of feature information at the front and back layers of the network. In addition, the network can mitigate network degradation, including gradient disappearance and explosion during training, which makes the model easier to converge and leads to stronger feature extraction capability. When the data set is large, the network model is enabled to have stronger learning ability and generalization ability so that the network model has better performance on classification tasks.

#### Coordinate Attention Shuffle Mechanism

Some of the images in the grape leaf data set that we collected were taken in a complex natural environment. The images have problems such as grape leaf self-obscuring, grape fruit, hand obscuring the disease area, leaf curling, and so forth. In addition, because some of the disease spots first occur at the edge of the leaf, the traditional method shows a large degree of uncertainty in terms of acquiring information about the grape leaf disease area. However, the gaps between different diseases on grape leaves are usually in tiny local details. If it is affected by both the background and the shape of disease spots at the same time, this will lead to increased recognition. In the current study, we found that CAM (Hou et al., 2021) can capture cross-channel information and orientation- and position-aware information, which can help the model locate and identify potential targets more precisely. Second, CAM is an attention method with flexible and lightweight properties that can be easily inserted into classic modules to enhance features by strengthening information representation. Finally, as a pre-trained model, CAM can bring significant gains to downstream tasks based on lightweight networks.

**FIGURE 5:**
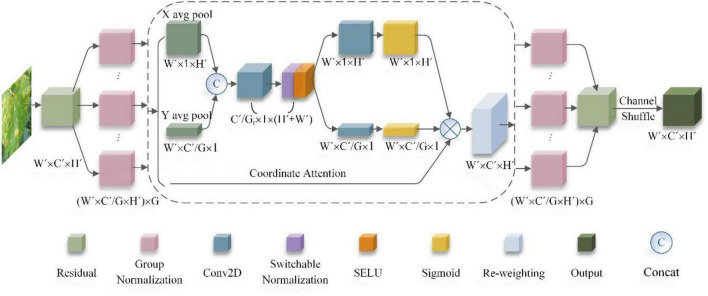
Coordinate attention shuffle mechanism (CASM) module structure.

Therefore, in this paper, we propose the CASM module, which is based on CAM, and added it to the CASM-AMFMNet so that the network model can pay closer attention to the grape disease area, distinguish the background interference from the disease, and accurately obtain the detailed feature information for the grape leaf disease area to extract the feature information to distinguish between diseases and improve recognition capability of the local detailed features of the disease. The CASM module is shown in Figure 5.

Because the CASM module is proposed according to the cam module, referring to the two steps of the cam module, this paper proposes that group coordinated information on the embedding module (GCM) and the coordinated attention generation shuffle module (CSM) are the main structures of the CASM module.

A. Group coordinate information embedding module (GCM)

This operation of the GCM corresponds to group convolution and the two parts X Avg Pool and Y Avg Pool in Figure 5 above, which is a global sensory field that encodes precise location information. The CASM module proposed in this paper has the following four improvements.

First, we GC the input image feature map of grape leaf disease. In network training, each sub feature map captures specific semantic information. After we performed GC, the parameter quantity became 1/G of the original standard convolution. With the increase in the number of groups, the parameter quantity and calculation quantity are significantly reduced. The G obtained by the experiment is set to 4. In addition, GC cannot easily produce overfitting, and it has the effect of regularization (Krizhevsky et al., 2017). Then, the attention module is induced to capture the remote dependencies with precise location information, and then the pooling kernel with dimensions (*H*, 1) and (1, *W*) is used to encode the sub-feature maps for each channel along the horizontal and vertical coordinates, respectively, so that the output of the *c*th channel can be written as the Equation (11).


(11) $$z_{c}=\frac{1}{H\times W}\,\sum_{j=1}^{H}\sum_{j=1}^{W}x_{c}\,\left(i,j\right)$$

In the Equation (11), *z*_*c*_ denotes the output of the *c*th channel and *x*_*c*_(*i,j*) denotes the values of the position characteristic diagram of height coordinate *i* and the width coordinate *j* of the *c*th channel. The output of the *c*th channel with height *h* can be expressed as the Equation (12).


(12) $$Z_{c}^{h}=\frac{1}{W}\,\sum_{0\leq j\leq W}x_{c}\,\left(h,j\right)$$

In the Equation (12), $Z_{c}^{h}\,\left(h\right)$ denotes the output with height of the *c*th channel as *h*, and *x*_*c*_(*h*,*j*) is the value of the feature map with width coordinate *j* for the *c*th channel with height *h*. The output of the *c*th channel with width *w* is as shown in the Equation (13).


(13) $$Z_{c}^{w}\,\left(w\right)=\frac{1}{H}\,\sum_{0\leq j\leq H}x_{c}\,\left(i,w\right)$$

In the Equation (13), $Z_{c}^{w}\,\left(w\right)$denotes the output with the height of the *c*th channel as *w*; *x*_*c*_(*i*,*w*) is the value of the feature map with height coordinate *i* for the *c*th channel with width *w*, and *H* and *W* are the height and width of the feature map, respectively.

The above two transformations aggregate features along two spatial directions and generate direction correlation feature graphs. This is very different from the SE block, which generates a single eigenvector in the channel attention method. These two transformations also allow the attention module to capture long-term dependencies along one spatial direction and preserve precise location information along the other, which helps the network to locate small spots more accurately.

B. Coordinate attention generation shuffle module (CSM)

In Step A, the global sensory field can be easily obtained, and precise positional information encoded. To better integrate the features of grape leaf diseases so that their features can be fully utilized to capture positional information and facilitate more precise localization of ROI regions, we concatenated the aggregation feature maps generated by Equations (12, 13), and we used 1*1 convolution to compress the channel for transformation to obtain Equation (14).


(14) $$f=\delta \,\left(F_{1}\,\left(\left[z^{h},z^{w}\right]\right)\right)$$

In Equation (14), [^*zh*^,^*zw*^] is a stitching operation along the spatial dimension, δ uses SELU, and *f* ∈ ^R*c*/*r*×(*H* + *W*)^ is an intermediate feature map that encodes the spatial information in the horizontal and vertical directions. Then, *f* is decomposed into two separate tensors ^*fh*^ ∈ ^R*c*/*r*×(*H* + *W*)^ and ^*fw*^ ∈ ^R*c*/*r*×(*H* + *W*)^ along the spatial dimension, and two additional 1*1 convolution transforms *f^h^* and *f^w^* are used to transform *F*_*h*_ and *F*_*w*_ into tensors with the same number of channels to the input X, respectively, to obtain Equations (15, 16).


(15) $$g^{h}=\sigma \,\left(F_{h}\,\left(f^{h}\right)\right)$$

(16) $$g^{w}=\sigma \,\left(F_{w}\,\left(f^{w}\right)\right)$$

In Equations (15, 16), σ is the sigmoid activation function to reduce the complexity and computational overhead of the model; an appropriate scaling ratio *r* is usually used here to reduce the number of channels of *f*. Next, the outputs *g*_*h*_ and *g*_*w*_ are expanded and used as attention weights to generate new feature maps by combining all of the sub-feature maps, as shown in Equation (17).


(17) $$y_{c}\,\left(i,j\right)=x_{c}\,\left(i,j\right)\times g_{c}^{h}\,\left(i\right)\times g_{c}^{w}\,\left(j\right)$$

Finally, by shuffling the information on the sub-feature map, we strengthened the information exchange between different channels and acted on the input to obtain the output *X* = [*x*_1_,*x*_2_,…*x*_*c*_] with the same size of this attention module as the input *Y*′ = [*x*_1_,*x*_2_,…*x*_*c*_] and with enhanced learning features, as shown in Equation (17).


(18) $$Y^{\prime }=c\,h\,a\,n\,n\,e\,l\,\_\,s\,h\,u\,f\,f\,l\,e\,\left(Y\right)$$

In GSM, the method used in this paper makes the following innovations.

1 To adapt to complex and variable backgrounds, we used switchable normalization (SN) instead of the traditional batch normalization (BN) layer to make the model more robust to adapt to various scenarios by dynamically adjusting the weights through training. SN calculates the BN, LN, and IN, produces the statistical weighting (weights are calculated by Softmax), and finally calculates the normalized pixel value _*h*⌢*nchw*_ as Equation (18).


(19) $${\overset{⌢}{h}}_{n\,c\,h\,w}=\gamma \frac{{\overset{⌢}{h}}_{n\,c\,h\,w}\sum_{k\,\varepsilon \,\Omega }\omega_{k}\,\mu_{k}}{\sqrt{\sum_{k\,\varepsilon \,\Omega }\omega_{k}^{\prime }\,\sigma_{k}^{2}+\varepsilon }}$$

In Equation (19), we input a four-dimensional feature vector of a grape leaf image with *n*, *c*, *h*, and *w*, representing the number of samples, channels, height, and width, respectively. *h*_*nchw*_ is each pixel on the feature map, _*h*⌢*nchw*_ is the pixel value output after the SN operation on*h*_*nchw*_, γ is the scaling coefficient; β is the offset coefficient, μis the mean value, σ^2^is the variance, and ω*_*k*_* and $\omega_{k}^{\prime }$ are the weighting coefficients for weighting the mean and variance, respectively. The weight coefficient ω*_*k*_* uses the Softmax function to calculate the control parameters λ*_*k*_* of the three dimensions, as shown in Equation (20).


(20) $$\omega_{k}=\frac{e^{\lambda_{k}}}{\sum_{z\,\varepsilon \,\Omega }e^{\lambda_{k}}}$$

In Equation (20), the initial values of the control parameters λ*_*k*_* for each of the 3 dimensions are 1, which are optimized during back propagation with _∑*k*εΩ_ω_*k*_ = 1; the value of each weighting factor ω*_*k*_* is between 0 and 1.

2 We used the SELU activation function instead of the commonly used ReLU activation function or the Sigmoid activation function to improve the learning convergence effect of the model. The SELU activation function is calculated as follows:


(21) $$S\,e\,L\,U\,\left(x\right)=\lambda_{s\,e\,l\,u}\,\left\{ \begin{array}{cc} x & x\geq 0\\ \alpha_{s\,e\,l\,u}\,\left(\exp\left(x\right)-1\right) & \text{otherwise} \end{array}\right.$$

In Equation (21), α and λ are hyperparameters, and it is proven that the training effect reaches the best atα_*selu*_≈1.6733,λ_*selu*_≈1.0507.

3 Adding a channel shuffle at the end of the module can effectively integrate the feature information on each channel, strengthen the information exchange between channels, and better enable the network fit of the extracted image features with fewer parameters to obtain higher model accuracy, improve the efficiency of the model operation, and enhance the classification effects.

#### Asymmetric Multi-Scale Fusion Module

Compared to the entire image, the diseased area on a grape leaf image is tiny, so the size of the disease spot used for the extraction itself is necessarily small. After CoAtNet, the semantic information of the small targets in the grape leaf features map almost disappears at this time, which increases the difficulty of the network to recognize small spots. The black rot spots and leaf bright spots are small and dense, the black measles spots are similar to stripes, and the frosty mildew spots are irregular in shape. To address the problem of small target recognition in grape leaves, we extracted and fused the shape and contour features of grape leaf spots at different scales, effectively improving network accuracy and enhancing the feature expression capability of the convolution kernel to achieve accurate recognition of small targets. A single-scale convolution kernel is not efficient for sensing multi-scale lesion points. Therefore, this paper proposes AMFM, which uses MSFM (Wang and Wang, 2020) as the framework for extracting the features of multi-scale lesions and partially improves it. AMFM can extract the small lesion features of grape leaves to a greater extent without increasing the amount of calculation and improve the robustness of the model to image reversal.

**FIGURE 6:**
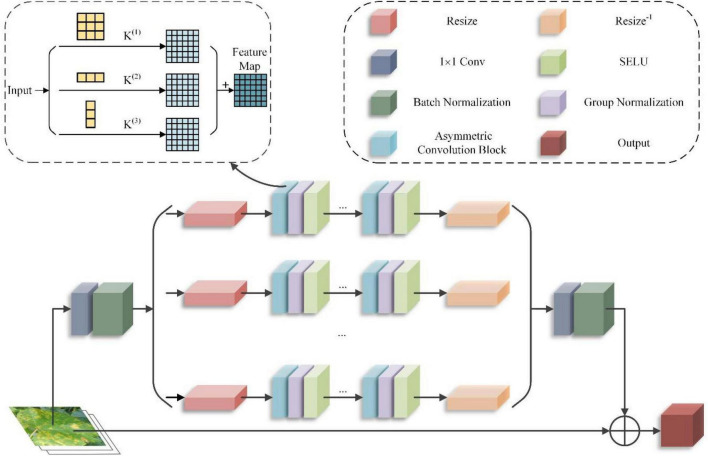
Asymmetric multi-scale fusion module (AMFM) structure.

Asymmetric multi-scale fusion module divides the feature map obtained after 1*1 convolution into n scales equally. One of the 3*3 convolutions is replaced using an asymmetric convolution block (ACB) (Ding et al., 2019), which can still extract features correctly for flipped images to improve the network’s training accuracy and reduce the parameters of the model training and the required computational effort. On the other hand, the use of the SELU activation function instead of the ReLU activation function can better fit the training, extract the features of the grape leaf spots, and improve the learning convergence of the network. The model for AMFM is shown in Figure 6.

First, the input grape leaf images are convoluted with 3*3 convolution kernels, 1*3 convolution kernels, and 3*1 convolution kernels, which produce three different shapes to extract different branch features, as shown in Equation (22). Then, the different branches are fused using convolution’s additivity to obtain the fused feature output. To make the module lightweight while maintaining the dimensionality of the fused output features consistent with that of the input features, the residual bottleneck structure is utilized. This structure refines the module input according to the channel and then feeds into the branches. Finally, the branch input is resized using bilinear interpolation, and its elements are returned to their original size using the same method, as shown in Equation (23).


(22) $$M\,\left(x\right)=x+U\,\left\{C\,\left[F_{1}\,\left(S\,\left(x\right)\right),F_{2}\,\left(S\,\left(x\right)\right),\ldots \,F_{n}\,\left(S\,\left(x\right)\right)\right]\right\}$$

(23) $$F_{n}\,\left(a\right)=R_{n}^{-1}\,\left(C\,G\,N_{n,i}\,\left(C\,G\,N_{n,i-1}\,\left(\ldots \,\left(C\,G\,N_{n,i}\,\left(R_{n}\,\left(a\right)\right)\right)\right)\right)\right)$$

In Equations (22, 23), x is the input grape blade, *M*(*x*) is the output, *S*() is the extrusion module that makes the input x thinner, *F*_*n*_() is the branching operation, *C*() is the combination function, and *U*() is the unsqueezed module that restores the branching output depth to be the same as *x*. *CGN*_*n*,i_ is the result of the extrusion module, *R*_*n*_() is the resize function on the nth branch, *a* = *S*(*x*) is the normalized nonlinear operation on the nth branch of the *i*th ACB group, and *R*_*n*_^–1^ is the resize function to restore the feature dimensions (height and width). The computational volume equation after applying the ACB is as shown in Equation (24).


(24) $$I*K_{1}+I*K_{2}=I*\left(K_{1}⊕K_{2}\right)$$

In Equation (24), *I* is the input feature map matrix; *K*_1_ and *K*_2_ are two convolution kernels; ⊕denotes added corresponding positions of the convolution kernels. In the feature fusion process of asymmetric convolution processing, the feature information is superimposed based on standard 3*3 convolution processing with feature information extracted by two dimensions of asymmetric convolution. Compared to the 3*3 convolution with 3*3 multiplications, the number of asymmetric convolution operations is 2*3 multiplications, and the amount of network operations is reduced by 1/3.

## Results and Analysis

This section verifies the effectiveness of the CASM-AMFMNet in the identification and classification of grape leaves through experiments and designs experiments to use the test set in other models together with the model in this paper to compare the effectiveness of different models. This section describes the experimental environment, the experimental setup, the evaluation metrics, the effectiveness analysis of each module of CASM-AMFMNet, the ablation experiments, and the comparison experiments between different models.

### Experimental Environment and Data Preparation

To verify the performance of the CASM-AMFMNet proposed in this paper, all experiments were carried out in the same hardware and software environment, with the specific environmental parameters shown in Table 2.

**TABLE 2:** Hardware and software environment.

Hardware environment	CPU	Intel Core i7-6800 K 3.40 GHz 15 MB
	RAM	64 GB
	Video memory	32 GB
	GPU	NVIDIA GTX 2080ti
**Software environment**	Operating system	Windows 10
	CUDA Toolkit	V11.1
	CUDNN	V8.0.4
	Python	3.8.8
	Torch	1.8.1
	Torch vision	0.9.1
	Matlab	2020a

### Experimental Settings

The self-made data set used in the experiments in this paper contained five categories of grape leaves: healthy, black rot, black measles, leaf blight, and downy mildew. The size of the unified image input is adjusted to 224*224 to improve the efficiency of the image processing technology, minimize the calculation cost, and reduce the time spent with the training model and classification. After pre-processing, we obtained a total of 15,824 images of grape leaves. The number of images for the five diseases was evenly distributed, all in the range of 19–21%. The data sets in this paper were divided in the following ratio: the training set: the validation set: the test set = 3:1:1, with 9,480 images of the five grape leaves in the training set and 3,160 images in the test set.

In the deep learning training, the hyperparameter selection is difficult and time-consuming because the optimal combination of hyperparameters depends not only on the model itself but also on the software and hardware environment. In this paper, the hyperparameters of the CASM-AMFMNet were determined through multiple fine adjustments, as shown in Table 3. When training with the model, batch training was adopted to randomly divide the training and validation sets into multiple batches, with a training batch (Minibatch) of 32 and a round batch (epoch) of 30 and 1,265 iterations per round, for a total of 37,950 iterations. We verified once every 1,000 iterations; the initial learning rate was set to 0.001, and the weight decay value was 1 × 10^–4^.

**TABLE 3:** Parameter setting.

Parameter category	Parameter name	Parameter setting
AdamW	Initial learning rate	0.001
	Weight decay	1 × 10^–4^
	Momentum	0.9
	Learning rate decay	0.1
Input data parameters	Size of input images	(224,224)
	Minibatch	32
	Iteration Epochs	30
	Iteration Number	37,950

To investigate the effects of different optimizers on model performance, three commonly used optimizers were selected for model training, and model accuracy was obtained under different optimizers. For validation accuracy, the AdamW (Kingma and Ba, 2014) optimizer value is 1.03 and 1.42% higher than those for the SGDM and RMSprop optimizers, respectively; for testing accuracy, the AdamW optimizer value is 1.71% and 2.10% higher than those for the SGDM and RMSprop optimizers, respectively. Therefore, the AdamW optimizer with the driving volume is more suitable for this study model. Under the same experimental conditions, the accuracy of the models obtained by the three optimizers differed significantly. Regarding training duration, the three methods are relatively close to one another, all occupying around 3 h, although the AdamW optimizer takes the shortest time.

Training parameters of the model are set as shown in Table 3.

### Individual Modules Effectiveness Analysis

#### Impact of Data Enhancement on Recognition Performance

**FIGURE 7:**
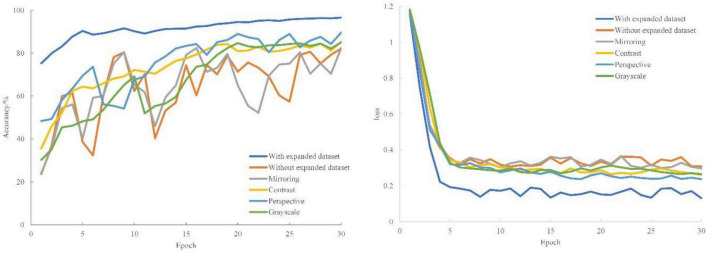
Comparison experiments of different data enhancement effects.

In this section, we used digital image processing to expand the collected grape leaf image data sets and then trained the original data set, the flip expanded data set, the contrast expanded data set, the gray expanded data set, the perspective expanded data set, and the common expanded data set using the model CASM-AMFMNet, proposed in this paper. The experimental results for accuracy and loss are compared to evaluate the impact of data enhancement on the classification accuracy of grape leaf diseases, as shown in Figure 7. Compared to the original image data set, the training accuracy of different expansion methods is improved by 0.30, 3.17, 2.86, 7.49, and 14.42 percentage points, respectively. The training accuracy in the case of the flipping expansion is not different from that in the original data set as the flipping operation shows little change in image quality due to multi-angle shooting. However, the training accuracy of other expanded data sets is significantly higher than that of the original data set. The reason for this is that the original training sample set is too small, and the data expansion provides the necessary amount of data for model training. In particular, the recognition accuracy of the jointly expanded data set is much better than for that of the non-expanded data set. The loss function curve shows that the training loss value of the expanded data set is lower, and the model converges rapidly; it can well fit the characteristics of grape leaf disease. Data expansion increases the diversity of data, the parameters of the classification model are fully trained, and the network model has better feature extraction ability when trained on large data sets. More importantly, the enhancement of the data set can better simulate the real environment of grape leaves and improve the model robustness.

#### Effectiveness of Gaussian Filters Sobel Smooth De-Noising Laplace Operator

To more objectively evaluate the feasibility of the method studied in this paper, the GSSL algorithm compares the grape leaf images processed by the GSSL algorithm with five filter enhancement and comparison algorithms, and the grape leaf image test set is enhanced for comparison experiments and analysis. The parameters of the gray level mean, peak signal-to-noise ratio (PSNR), and entropy of the six algorithms are shown in Table 4.

**TABLE 4:** Enhanced image quality parameters.

Method	Mean	PSNR	Entropy
Original image	132.17	27.49	7.55
EGIF (Wu et al., 2021)	135.91	29.63	7.64
WGIF (Mu et al., 2021)	110.23	31.08	7.25
HSFGTF (Joseph et al., 2021)	128.25	28.70	7.00
GFCBH (Pashaei, 2021)	95.16	35.12	6.99
WLS (Singh et al., 2022)	119.32	35.04	7.81
GSSL	148.61	37.87	7.94

From the data obtained in Table 4, it can be observed that the grape leaf image derived from this experiment is significantly improved relative to the original image. The PSNR of the enhanced image obtained by this method is 8.24, 6.79, 9.17, 2.75, and 2.83 dB higher than those for EGIF, WGIF, HSFGTF, GFCBH, and WLS, respectively, and 10.38 dB higher than that of the original image, indicating that the image enhanced with the algorithm used in this paper has less distortion and higher quality; the obtained entropy values of the enhanced image are 0.3 bit, 0.69 bit, 0.94 bit, 0.95 bit, and 0.13 bit larger than those for other methods and 0.39 bit larger than that of the original image, resulting in improved image quality and a greater amount of information. At the same time, the means of the enhanced image in this paper are 12.7, 38.38, 20.36, 53.45, and 29.29 higher than those of other methods and slightly higher than that of the original image and 16.44 higher than that of the original image, which makes the enhanced image brighter and more appreciable. The extraction of image edge details at the same time ensures the authenticity of the image information, effectively overcomes the impact of noise in the image, and makes the leaf details clearer, the image brighter, and the grape leaf spot texture obvious and readable.

#### Self-Contrasting Experiments

We carried out self-comparison experiments on the grape leaf dataset for the underlying network CoAtNet model and our CASM-AMFMNet network model. First, the training set is used for training, and then the obtained model is tested against the test set. By contrast, the network model in this paper shows some improvement in recognition speed and accuracy compared to the CoAtNet network model.

**FIGURE 8:**
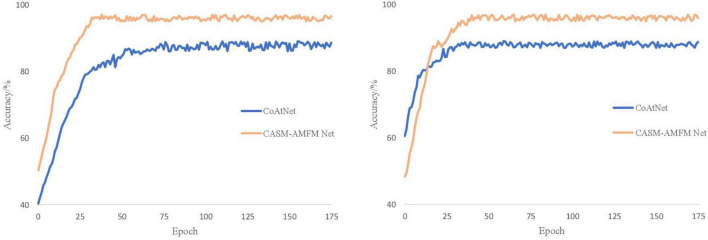
Accuracy curves of the CoAtNet and the coordinated attention shuffle mechanism-asymmetric multi-scale fusion module net (CASM-AMFMNet).

It can be seen from Figure 8 that CoAtNet iterations tend to converge 50 times, and the final training accuracy is 88.56%, while CASM-AMFMNet iterations tend to converge 30 times, and the final training accuracy is 96.58%, which is higher than CoAtNet. Because Sn and GN are added to the CASM-AMFMNet algorithm, the convergence speed of the model may accelerate. The accuracy rate for the CoAtNet test set is 88.74%, and it is 95.95% for the CASM-AMFMNet test set. Because the proposed algorithm incorporates contextual and location information among the grape leaf disease regions, the accuracy rate on the test set is 7.21% higher than that of CoAtNet.

It can be seen from Table 5 that the number of parameters of our improved CASM-AMFMNet network is much smaller than the number from before the improvement (−2 M). In addition, ACB divides the standard convolution into 1*3 and 3*1, which further reduces some parameters of the original convolution layer and greatly improves the overfitting-prone characteristics of the complex network. When the number of parameters is reduced, the recognition accuracy is improved (+10.78%). In terms of program running time, with 32 samples per training batch, the original model takes 586 s, while the improved model takes only 270 s (−316 s). The difference in the total program time is even more obvious, with a 109.61-min difference in the time spent to train 30 epochs, reflecting the improved performance of the updated model in terms of the training cost. Our improved model significantly lowered the number of parameters. Its effectiveness is reflected not only in preventing overfitting and thus improving test accuracy but also in the time cost required for the training, which is highly practical.

**TABLE 5:** Performance comparison of CoAtNet and coordinated attention shuffle mechanism-asymmetric multi-scale fusion module net (CASM-AMFMNet).

Method	CoAtNet	CASM-AMFMNet
Accuracy	88.74%	95.95%
mAP	79.49%	90.27%
FPS	44	85
Param	168 M	166 M
FLOPs	189.5 B	187.8 B
MFLOPs	632.79 MB	4.67 MB
Running time per batch	586 s	270 s
Time required per epoch	169.86 min	60.25 min

#### Backbone Comparison Experiment

To determine the choice of the model backbone in this paper, under the framework of CASM-AMFMNet, models with backbone from CoAtNet −1 to 7 are experimentally compared. The experimental results are shown in Table 6.

**TABLE 6:** Experimental results for different backbone networks.

Models	Eval size	Params	FLOPs	Accuracy
CoAtNet-1	224^2^	55 M	49.8 B	89.56%
CoAtNet-2	224^2^	75 M	96.7 B	92.45%
CoAtNet-3	224^2^	96 M	126.1 B	93.98%
CoAtNet-4	224^2^	121 M	149.8 B	94.77%
CoAtNet-5	224^2^	166 M	187.8 B	95.95%
CoAtNet-6	224^2^	275 M	289.8 B	95.98%
CoAtNet-7	224^2^	330 M	360.9 B	96.01%

When the size of the grape leaf image data set is the same, the width of the network increases with the increase in the CoAtNet model. The network params and flops between CoAtNet −1 and 5 are very small, but the recognition accuracy differs by more than 1 percentage point. From CoAtNet −5, the recognition accuracy improves slightly. Where the depth of the network layer is deepened, CoAtNet −6 and CoAtNet −7 increase the accuracy of the network model weight file by 0.03 percentage points after 109 M and 0.06 percentage points after 164 M. There is little difference in the recognition accuracy among CoAtNet −6, CoAtNet −7, and CoAtNet −5. Therefore, CoAtNet −5 with a moderate size of params and flops and a high recognition accuracy for the grape leaf image data set is used in all subsequent experiments.

#### Effectiveness of Coordinate Attention Shuffle Mechanism

To verify the effectiveness of the CASM module, we first experimented with the settings of the grouping parameter G (see a below), and then verified the effects of the activation function, shuffling strategy, and the attention mechanism in the CASM module on the model through three experiments (see b–d below).

(a) Effects of different grouping numbers on the CASM module. The grouping number G is set to 2, 4, and 8. The analysis data are grape leaves, and the results of the analysis of different parameters are shown in Table 7.

**TABLE 7:** Comparison result of different groups.

Group number	Test accuracy	mAP	Testing time	Params	Flops
G = 2	95.95	90.25%	11.33	166.63 M	189.5 B
G = 4	95.95	90.27%	10.87	166.41 M	188.9 B
G = 8	95.95	90.23%	11.83	166.30 M	188.6 B

As shown in Table 7, the test accuracy of the model is close to 90% for all three cases, with a different number of groups. With the increase in the number of groups, the params (−0.22 M, −0.11 M) and flops (−0.6 B, −0.3 B) of the network model are significantly reduced. Although the increase in the number of groupings reduces the computational and parametric quantities of the model, its intensive operations lower the computing and storage access efficiency and extend the actual running time. Therefore, in practical applications, combining the above reasons and experimental data, we set the number of groups of CASM to G = 4.

(b) The influence of SN and SELU activation functions on the model is used in the CASM module. To verify the feasibility and effectiveness of SN and SELU, this paper validates them in CoAtNet in terms of training time and training accuracy with different batching methods and activation functions, and the results are shown in Table 8.

**TABLE 8:** Exploring the combination of normalized processing and activation functions.

Method	mAP	Param	Training time
BN+ReLU	80.31%	167.33 M	4 h 48 min 29 s
BN+Sigmoid	79.85%	167.95 M	4 h 58 min 57 s
BN+SELU	81.07%	166.53 M	4 h 16 min 42 s
SN+ReLU	80.91%	167.35 M	4 h 50 min 03 s
SN+Sigmoid	80.45%	167.97 M	4 h 59 min 44 s
SN+SELU	81.67%	166.55 M	4 h 18 min 09 s

In Table 8, the SN + SELU combination is shown to be better than BN/SN + ReLU, BN/SN + Sigmoid, and BN + SELU in mAP, with increases of +1.36%, +0.76%, +1.91%, + 1.31%, and +.69%, respectively, and compared to the most common BN + ReLU combination; its param is also reduced by about 0.78 M, and the training time is reduced by 30 min and 20 s, which makes the optimization learning and solving model convergence easier.

(c) The CASM module introduces the effects of channel shuffling strategies on the model. The grouping of grape leaf features generates a large amount of group convolutional stacking, which leads to feature information loss and the obstruction of the interactive flow of feature information between channels, as well as seriously affecting the feature characterization ability. In this paper, we introduce a channel-mixing strategy into the proposed module and compare the same module without adding the mixing operation to verify the impact of adding channel mixing on grape leaf disease identification. The model uses CoAtNet as the backbone network for comparative analysis of channel-mixing additions in the framework of CASM-AMFMNet.

The experimental results in Table 9 indicate that the inclusion of the shuffling operation in the model does not generate additional parameters or computational effort, and the presence of the shuffle channel was effective in improving the average identification accuracy of grape leaf diseases. The use of channel shuffling after all convolutional layers using grouped convolution improves the accuracy of the model by 0.34 percentage points. Channel shuffling enhances the flow of feature information between channels, and plays a positive role in the interaction of feature information obtained from group convolution, which makes the model more efficient in its use of feature information in different channels that are at the same spatial location after group convolution, improving the experimental accuracy.

**TABLE 9:** Effect of shuffle on the model.

	CASM-AMFMNet (no Shuffle)	CASM-AMFMNet (with Shuffle)
mAP	89.93%	90.27%
FLOPs	189.5 B	189.5 B
param	166 M	166 M

**FIGURE 9:**
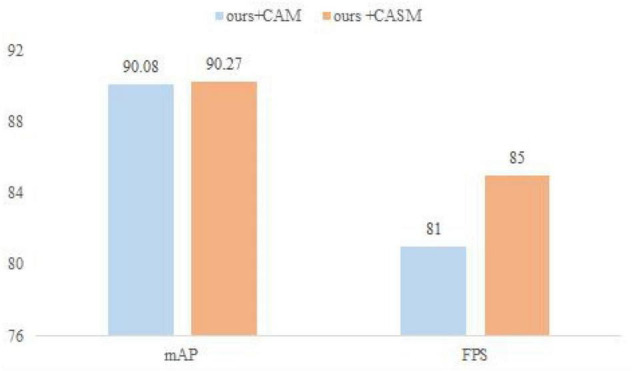
Effects of different attention modules of coordinate attention shuffle mechanism (CASM) and CAM on model recognition ability.

(d) The CASM module uses the attention module for the impact on the model. While accomplishing network light weighting, channel attention is particularly important for ensuring network accuracy. This set of experiments is conducted to verify the effect of the CAM and CASM modules on the model, as well as the effect of different positions and quantities of CASM on the grape leaf disease data set. Under the same training environment, the performance of different attention modules of the CASM module and CAM on the model recognition ability is shown in Figure 9. The CASM module was added at different positions of CASM-AMFMNet to study the correlation between the recognition ability of the model and increasing the number of CASM attentions. The experimental results are shown in Table 10.

**TABLE 10:** Experimental results of adding an attention mechanism to different positions and numbers.

Location	Number	mAP	FLOPs
Add CASM module to CoAtNet	×1	89.62%	189.5 B
	×2	89.45%	190.1 B
Add CASM module after CoAtNet	×1	90.27%	189.5 B
	×2	90.10%	190.1 B
Add CASM module after AMFM	×1	90.03%	189.5 B
	×2	89.86%	190.1 B

As can be seen in Figure 9, for the data set in this paper, comparing the CASM module and CAM, the mAP and FPS of the CASM module are higher than those of CAM. Therefore, this paper uses the CASM module to fuse with the feature extraction network. From Table 10, we can see that the feature blending effect generated by redundant features has an impact on extraction accuracy, and the use of the attention mechanism also generates additional computational overhead, and the complexity of the network model increases with the number of CASM modules inserted (+0.6 B). Therefore, after experimental comparison, adding the CASM module after the CoAtNet module gives the best experimental results and an accuracy of 90.27%, which is 0.65 and 0.24% higher than the accuracy of the other two models. In this paper, the CASM attention module is embedded into the feature extraction network, which has the effect of suppressing invalid leaf and background features and enhancing effective grape leaf disease features, as well as improving the performance of correctly capturing the disease location information of grape leaf feature extraction network.

#### Effectiveness of Asymmetric Multi-Scale Fusion Module

To study the effectiveness of each part of AMFM on the grape leaf data set, this paper takes CoAtNet as the backbone, and AMFM is used as a single ablation experiment on the grape leaf data set, i.e., comparing MSFM, replacing 3*3 convolution of MSFM with ACB, replacing RELU of MSFM with SELU, and using AMFM to perform the experiment, with the experimental results shown in Table 11.

**TABLE 11:** A single ablation experiment of asymmetric multi-scale fusion module (AMFM).

Method	mAP	Params	FLOPs	Testing time
CoAtNet with MSFM	82.10%	169.31 M	191.0 B	35.41 s
CoAtNet with MSFM (ACB)	83.44%	168.98 M	190.3 B	33.33 s
CoAtNet with MSFM (SELU)	84.03%	168.85 M	189.9 B	32.08 s
CoAtNet with AMFM	85.37%	168.52 M	189.2 B	29.13 s

As seen in the experimental results given in Table 11, the AMFM proposed in this paper has a significant effect on the improvement of network identification performance. After adding AMFM to the CoAtNet network, the mAP is increased by 3.27%, the params are reduced by 0.79 M, the FLOPs are reduced by 0.8 B, and the test time is reduced by 6.28 s relative to adding MSFM, which indicates that the use of ACB and SELU in the AMFM can significantly reduce the number of params and the number of operations and improve the network training efficiency. When the traditional standard convolution in MSFM is experimentally replaced with ACB, the accuracy of using ACB is slightly improved compared to the traditional convolution (+1.34%), the params decrease by 0.33 M, the FLOPs decrease by 0.7 B, and the test time decreases by 2.08 s, which indicates that ACB can improve the performance of the underlying model. When ReLU is replaced with SELU in MSFM, mAP increases by 0.93%, params decrease by 0.46 M, FLOPs decrease by 1.1 B, and test time decreases by 3.33 s, which shows that the activation function SELU can better improve the convergence speed and recognition accuracy of the model compared to ReLU. The experimental results fully demonstrate the effectiveness of the AMFM proposed in this paper and further enhance the richness and representation capability of the features extracted by the model. In addition, adding the module further improves the results of grape leaf disease recognition in several comparative experiments.

### Ablation Experiments

**FIGURE 10:**
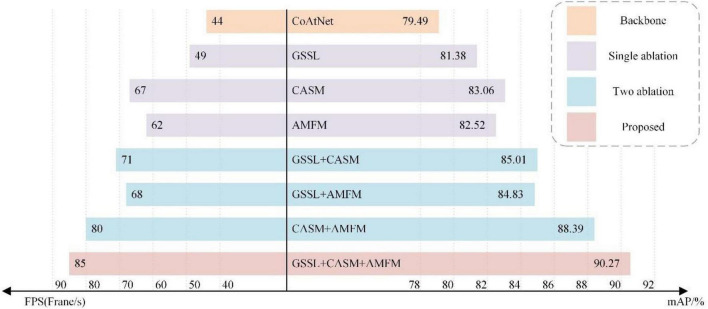
Ablation experiments.

To verify the effectiveness of the CASM-AMFMNet, ablation experiments are conducted on the proposed CASM-AMFMNet network for the grape leaf image data set. Taking CoAtNet as the backbone, to which GSSL, CASM, and AMFM are gradually added, the performance of each module is analyzed by comparing the differences in detection accuracy and FPS. The overall ablation experiments are shown in Figure 10.

As can be seen from the ablation experiments, the model performance of the GSSL algorithm on the basis of the backbone improves by about 1.89% in mAP and about five in FPS. After adding only CASM, the map quality increases by about 3.57% in mAP and about 23 in FPS; adding AMFM alone increases it by about 3.03% in mAP and about 18 in FPS. To sum up, the CASM-AMFMNet increases 10.78% in mAP and increases in recognition speed (+41) compared to CoAtNet. The above seven sets of experimental results demonstrate the effectiveness of GSSL, CASM, and AMFM. This illustrates the high accuracy and speed of the network used in this paper for the identification of grape leaf diseases.

### Comparison of Coordinated Attention Shuffle Mechanism-Asymmetric Multi-Scale Fusion Module Net With Other Classification Models

**FIGURE 11:**
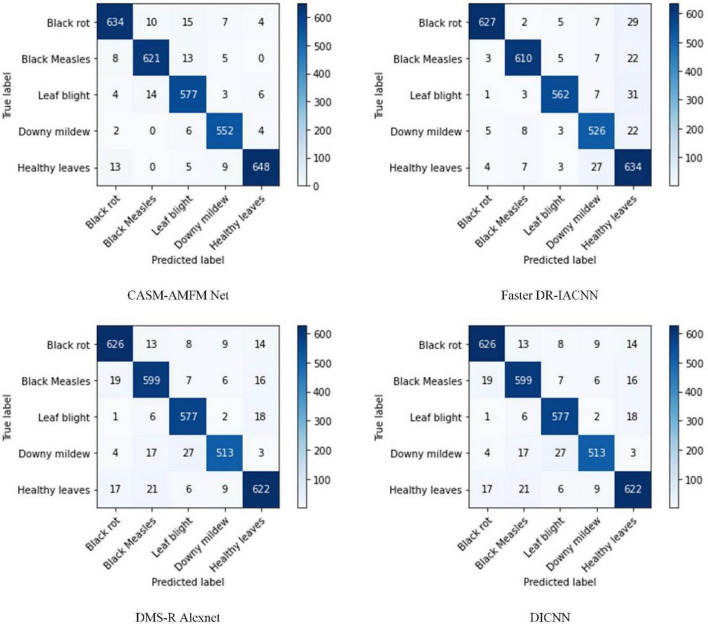
A confusion matrix for the identification of grape leaf diseases.

Overall, 670 black rot, 647 black measles, 604 leaf blight, 564 downy mildew, and 675 healthy grape leaf images were selected as a fixed test dataset. All images in the dataset were not involved in the training of the model. Therefore, the generalization ability of the model was tested based on recognition accuracy, i.e., whether the model had the same high recognition accuracy for grape leaf images not involved in training. The performance of CASM-AMFMNet was further compared with the other three networks using the confusion matrix, as shown in Figure 11. Diagonal cells in the confusion matrix indicate the number of test samples correctly predicted by the model and non-diagonal cells the number of samples incorrectly predicted by the model.

Through the confusion matrix, we identified that, among the 670 black rot test samples of our CASM-AMFMNet network, four were incorrectly identified as healthy grape leaves, 10 as black measles, and 15 as leaf blight. Among the 604 leaf blight samples, only four were wrongly identified as healthy leaves and 14 as black measles. Six of the 564 downy mildew samples were incorrectly identified as healthy leaves and 13 of the 647 black measles test samples as healthy leaves. During classification of the four major diseases and healthy grape leaves, on the one hand, since disease spots of black rot and leafy blight were too small and limited in number at the early stage of the onset, similar to the images of healthy leaves, errors inevitably occurred in the identification of categories. On the other hand, misidentification phenomena were commonly encountered. (1) Black rot and leaf blight were easily misclassified as black measles due to connections in the later disease stages. Specifically, shape characteristics were similar to black measles, and color differentiation was not high, leading to classification errors. (2) Black rot manifested as small brown spots at the beginning, which could easily be confused with leaf blight. (3) Black measles presented as long brown spots on the leaf surface and leaf blight showed similar characteristics to black measles in terms of spot color, shape, and texture at the margins of the grape leaf surface. However, downy mildew differed significantly from the other three diseases in terms of both color and shape characteristics, resulting in a higher recognition rate. Among the five categories of grape leaves, 3,032 were correctly identified and classified with our network model, 2,959 with Faster DR-IACNN, 2,937 with DMS-R Alexnet, and 2,957 with DICNN. Based on the comparison data, we conclude that our newly developed network model has the highest feature recognition and classification efficiency.

Accuracy, precision, recall, and F1 scores of the four network models for four grape leaf diseases and healthy grape leaves were calculated using the confusion matrix as the model performance evaluation index (Table 12).

**TABLE 12:** Performance evaluation of four types of networks.

Class	Evaluation metrics	Black rot	Black measles	Leaf blight	Downy mildew	Healthy leaves	Average value
DICNN	Accuracy	97.31%	96.68%	97.63%	97.56%	96.71%	97.18%
	Precision	93.28%	94.44%	93.71%	92.73%	93.63%	93.56%
	Recall	94.27%	95.32%	96.75%	97.21%	86.22%	93.95%
	F1 Score	93.77%	94.88%	95.21%	94.92%	89.77%	93.71%
DMS-R Alexnet (Lv et al., 2020)	Accuracy	97.31%	96.68%	97.63%	97.56%	96.71%	97.18%
	Precision	93.43%	92.58%	95.53%	90.96%	92.15%	92.93%
	Recall	93.85%	91.31%	92.32%	95.18%	92.42%	93.02%
	F1 Score	93.64%	91.94%	93.90%	93.02%	92.28%	92.96%
Faster DR-IACNN	Accuracy	98.23%	98.20%	98.16%	97.28%	95.41%	97.46%
	Precision	93.58%	94.28%	93.05%	93.26%	93.93%	93.62%
	Recall	94.71%	96.06%	97.91%	95.46%	85.91%	94.01%
	F1 Score	94.14%	95.16%	95.42%	94.35%	89.74%	93.76%
CASM-AMFM Net (ours)	Accuracy	98.01%	98.42%	97.91%	98.86%	98.70%	98.38%
	Precision	94.63%	95.98%	95.53%	97.87%	96.00%	96.00%
	Recall	95.92%	96.28%	93.67%	95.83%	97.89%	95.92%
	F1 Score	95.27%	96.13%	94.59%	96.94%	95.96%	95.78%

As shown in Table 12, the average accuracy of the model was 98.38%, which was 0.92, 1.2, and 1.2% higher than that of Faster DR-IACNN, DMS-R Alexnet, and DICNN, respectively. The average precision rate of 96.00% was 2.38, 3.07, and 2.44% higher relative to the above three models, respectively. Average recall value was 95.92%, which was 1.91, 2.9, and 1.97% higher, and the average F1 score of 95.78% was 2.05, 2.82, and 2.07% higher compared to the other three models, respectively. In summary, our model shows good recognition accuracy for grape leaf diseases.

To further validate the effectiveness of our model, the methods used by other researchers to resolve the image recognition problem of plant leaf datasets were introduced for comparison. Overall, 10 deep network models were selected, and experimental results are shown in Table 13.

**TABLE 13:** Comparison of the main performance of different methods.

Method	A	P_*A*_	R_*A*_	F1_*A*_	mAP	Training time
DCNN (Ma et al., 2021)	83.87%	84.73%	81.29%	82.97%	80.77%	4 h 04 min 12 s
MediNET (Bhuiyan et al., 2021)	76.99%	76.83%	77.29%	77.06%	78.39%	5 h 58 min 27 s
YoloV4 (Richey and Shirvaikar, 2021)	63.42%	59.21%	68.35%	63.45%	71.29%	3 h 6 min 45 s
VirLeafNet (Joshi et al., 2021)	85.12%	84.54%	77.87%	81.06%	81.73%	4 h 28 min 03 s
BGCNN (Hridoy and Rakshit, 2022)	91.59%	91.20%	91.00%	91.10%	84.44%	3 h 32 min 57 s
DCGAN (Zhao et al., 2021)	83.79%	82.31%	83.54%	82.92%	85.89%	4 h 38 min 27 s
OPNN (Akanksha et al., 2021)	82.38%	81.16%	83.28%	82.21%	81.23%	3 h 50 min 3 s
DICNN	93.58%	93.56%	93.95%	93.71%	84.81%	3 h 30 min 51 s
DMS-R Alexnet	92.94%	92.93%	93.02%	92.96%	85.84%	4 h 26 min 9 s
Faster DR-IACNN	93.64%	93.62%	94.01%	93.76%	87.48%	3 h 48 min 13 s
CASM-AMFM Net(ours)	95.95%	96.00%	95.92%	95.78%	90.27%	3 h 13 min 27 s

As evident from Table 13, YoloV4 and MediNET had relatively low recognition accuracy of <80% for grape leaf disease images. The two networks are less focused on the context and location information between disease regions in the recognition process, and, therefore, recognition effects are poor. Accuracy levels of DCNN, VirLeafNet, DCGAN, and OPNN in the test set were estimated as 83.87, 85.12, 83.79, and 82.38%, respectively, which have a deeper network structure and can extract deep-seated grape leaf disease features but still do not consider the contextual and location information among disease regions. The algorithm proposed in this study incorporating contextual and location information among disease regions achieved 95.95% accuracy on the test set, which is higher than all the other network models examined (BGCNN, DICNN, DMS-R Alexnet, Faster DR-IACNN), with >90% accuracy (+4.36, +2.37, +3.01, +2.31%, respectively). CASM-AMFMNet also achieved more accurate localization compared to the training time required for CASM-AMFMNet and other deep models. The training time required for CASM-AMFMNet for grape leaf images was 3 h 13 min 27 s, which was significantly lower than the time taken by other models. Our findings clearly demonstrate enhanced recognition performance and robustness of CASM-AMFMNet developed in this study in terms of training time, recognition accuracy, precision, recall, and F1 score.

## Discussion

Here, we constructed a CASM-AMFMNet model capable of effectively extracting shape, color, and texture features of grape leaf images to automatically improve identification and classification of healthy and diseased leaves. Application of the model to grape leaf images from the public PlantVillage Dataset (Kaggle, 2019) led to the recognition of four types of grape leaf diseases and healthy grape leaves with an accuracy of 97.21%. We further applied this novel model to the self-made banana leaf image dataset collected from Guangdong Province along with leaf images of apple, corn, and cherry from the PlantVillage dataset. The average classification accuracy of different diseases of the leaves from various plant species reached 94.41, 96.09, 94.77, and 95.92% for banana, apple, corn, and cherry, respectively. Comparative analysis suggested that the actual effect of these kinds of blades using our model is inferior to that of other methods for the above blades, and accuracy is additionally lower. Overall, the accuracy of our CASM-AMFMNet model in identifying grape leaf diseases was greatly enhanced compared with the other leaf types, and its classification effect was superior. As the shape of grape leaf edges is not a regular oval, the majority of disease spots are water stains and the edge contours are obvious. The color of disease spots is clearly distinct from that of the leaf surface, which is not observed for other leaf types.

The accuracy of grape leaf disease identification with the CASM-AMFMNet network was significantly higher than that with existing methods and solved the problem of low accuracy of multi-classification grape leaf disease identification to a certain extent, but further studies are necessary to resolve a number of issues. (1) The model training speed could potentially be reduced through more advanced parallel processing. (2) At present, we are limited to extraction and identification of the characteristics of only single grape leaf diseases using this method. Features that could enhance identification of two or more similar mixed diseases (Barbedo, 2016) or other diseases of grape leaves require further investigation. (3) Grape leaf contours are valuable for studying disease types, and methods to segment out the disease spots and leaf contours will be a focus of future research. The algorithm proposed in this study still needs further fine-tuning to improve the recognition rate of diseases from images with inconspicuous features. In particular, leaf features at the early onset of the grape disease onset are inconspicuous, and some disease characteristics are more similar, resulting in low recognition rates at the early stages of infection.

## Conclusion

To address the challenges of identification of grape leaf diseases, which are easily confused with the background, and difficulty of detection of small spots under complex backgrounds, we first constructed a dataset for grape leaf disease target recognition and classification, comprising a total of 15,824 images. Next, the GSSL algorithm was used to enhance the texture of grape leaves on the original image. After processing, this technique increased the map of the network by 1.89% and FPS by five. We further applied the CASM-AMFMNet model, which reduced the background interference in feature extraction without segmenting the background of grape leaves. The CASM-AMFMNet model was improved based on CoAtNet. The CASM module captured and pinpointed leaf diseases and effectively prevented confusion with the background, following which AMFM facilitated the identification of smaller target spots, which improved model recognition performance to a greater extent. Addition of CASM to CoAtNet increased mAP by 3.57% and FPS by 23, and adding AMFM to CoAtNet increased mAP by 3.03% and FPS by 18. Overall, CASM-AMFMNet was effective in identifying four grape diseases, specifically black rot, black measles, leaf blight, and downy mildew, with 98.01, 98.42, 97.91, and 98.86% accuracy, respectively, and healthy grape leaves with 98.7% accuracy. The average recognition accuracy of the five categories of grape leaves was >98%. Our collective results demonstrate enhanced performance of CASM-AMFMNet in identifying grape leaf spots and diseases with good accuracy and speed.

The CASM-AMFMNet model can be successfully applied for real-time disease identification from images of grape crop leaves under complex backgrounds, which is crucial for timely diagnosis and control of foliar pests and diseases that affect cultivated grape vines. In future studies, we plan to focus on application of the model to identify more leaf disease types and further improve the network by enhancing the feature extraction ability, reducing the recognition time and increasing accuracy. In addition, we will consider the transplantation of this model to cell phone platforms to enable more effective immediate identification of grape leaf diseases for raising agricultural productivity.

## Data Availability Statement

The original contributions presented in the study are included in the article/supplementary material, further inquiries can be directed to the corresponding author.

## Author Contributions

JS: methodology, writing–original draft preparation, conceptualization, and data curation. JZ: software, data acquisition, and investigation. GZ: validation and project administration. AC: supervision and funding acquisition. YoH: software. WH: writing, review, and editing. WC: model guidance. YhH: formal analysis and resources. LL: visualization. All authors contributed to the article and approved the submitted version.

## Conflict of Interest

The authors declare that the research was conducted in the absence of any commercial or financial relationships that could be construed as a potential conflict of interest.

## Publisher’s Note

All claims expressed in this article are solely those of the authors and do not necessarily represent those of their affiliated organizations, or those of the publisher, the editors and the reviewers. Any product that may be evaluated in this article, or claim that may be made by its manufacturer, is not guaranteed or endorsed by the publisher.
